# Purification and Characterization of Resistant Dextrin

**DOI:** 10.3390/foods10010185

**Published:** 2021-01-18

**Authors:** Yuanhang Zhen, Tao Zhang, Bo Jiang, Jingjing Chen

**Affiliations:** 1 State Key Laboratory of Food Science and Technology, Jiangnan University, Wuxi 214122, China; 6180112163@stu.jiangnan.edu.cn (Y.Z.); zhangtao@jiangnan.edu.cn (T.Z.); jingjinc@jiangnan.edu.cn (J.C.); 2 International Joint Laboratory on Food Safety, Jiangnan University, Wuxi 214122, China

**Keywords:** resistant dextrin, purification, membrane filtration, anion exchange resin, characterization

## Abstract

In this study, an efficient method for the purification of resistant dextrin (RD) using membrane filtration and anion exchange resin decolorization was developed, then the purified RD was characterized. In the membrane filtration stage, suspended solids in RD were completely removed, and the resulting product had a negligible turbidity of 2.70 ± 0.18 NTU. Furthermore, approximately half of the pigments were removed. Static decolorization experiments revealed that the D285 anion exchange resin exhibited the best decolorization ratio (D%), 84.5 ± 2.03%, and recovery ratio (R%), 82.8 ± 1.41%, among all the tested resins. Under optimal dynamic decolorization conditions, the D% and R% of RD were 86.26 ± 0.63% and 85.23 ± 0.42%, respectively. The decolorization efficiency of the D285 resin was superior to those of activated carbon and H_2_O_2_. Moreover, the chemical characteristics and molecular weight of RD did not change significantly after purification. The nuclear magnetic resonance spectroscopy of RD showed the formation of new glycosidic linkages that are resistant to digestive enzymes. The superior water solubility (99.14%), thermal stability (up to 200 °C), and rheological properties of RD make it possible to be widely used in food industry.

## 1. Introduction

Resistant dextrin (RD) is a water-soluble dietary fiber that is made of pyrodextrin after a certain degree of hydrolysis using α-amylase or glucoamylase [1]. During dextrinization, starch is degraded under the action of acid and heat, and new bonds, including β-1,6, β-1,2, α-1,6, and α-1,2 bonds, are formed. The branched structure of RD is considered responsible for its increased resistance to digestion [2]. RD is of physiological importance as it helps in controlling intestinal functions and moderating the adsorption of sugar and neutral fat after a meal. Therefore, RD is widely utilized in healthy foods [3].

Unfortunately, many impurities are generated during the production of RD. Under the action of enzymes, some of the pyrodextrin is degraded into monosaccharides, thus affecting the purity of RD. In addition, many colored compounds are produced as a consequence of the Maillard reaction and caramelization when heating starch in addition to liquefying and saccharifying pyrodextrin during the production process. Golon et al. [4] analyzed the product ingredients obtained from starch through pyrodextrin. In these thermal treatments processes, all carbohydrates undergo significant chemical changes frequently described as nonenzymatic browning reactions. The best-known examples are the thermal conversion of glucose and sucrose to caramel and the Maillard reaction between saccharides and amino acids at elevated temperatures, forming materials referred to as melanoidins. The colored substances are mainly aromatic compounds [5]. Three types of pyrodextrins, namely white dextrin, British gum, and yellow dextrin, are prepared under different conditions, and they have different colors such as white and brown [6]. High temperature and low pH during the preparation of yellow dextrin lead to the formation of more indigestible ingredients. In addition, yellow dextrin has an excellent solubility of up to 95–100%. However, higher amounts of brown pigments are also produced [7], suggesting that yellow dextrin is not suitable for use in the food and pharmaceutical industries. Therefore, it is vital to decolorize yellow dextrin for extensive applications in dietary supplements and medical practice.

Membrane processes have been widely utilized for over four decades in the food industry [8]. Depending on the particle size retained, filtration can be classified as microfiltration (MF), ultrafiltration (UF), nanofiltration (NF), or reverse osmosis (RO). In fact, membrane technology has been extensively used in sugar refining, such as the purification and decolorization of molasses and the refining of re-melted raw sugar [9,10]. Vu et al. used UF membrane technology to clarify sugarcane; this technology was applied as a substitute for chemical purification [11]. Dirt, especially proteins and other macromolecules, can cause fouling by entering both membrane surfaces and membrane pores. It was found that the main fouling mechanism of ceramic membranes with pore sizes less than a certain value involved the cake layer. For membranes with a pore size larger than this value, the fouling mechanism involves pore blocking [12]. The pH has a significant impact on the flux. Susanto et al. found that the flux under acidic conditions (pH = 4) declined more dramatically compared to that under neutral or alkaline conditions, suggesting that more fouling occurred under acidic conditions. This is because a decrease in the solution pH is accompanied by protonation, which decreases the electrostatic repulsion force between the membrane surface and the feed component [13]. Based on the previous research [13], we chose a UF membrane (5 kDa pore size) and an NF membrane (300–500 Da pore size) for the separation step. However, the experimental results indicated the presence of low-molecular-weight pigments that could not be retained by the membrane. Therefore, further processing of the permeate by ion exchange resins or other adsorbents (such as activated carbon) is necessary for complete decolorization [14].

Various approaches such as H_2_O_2_ oxidation [15,16], activated carbon adsorption [17], and ion exchange technology [18] have been used for decolorization. However, H_2_O_2_ oxidation may result in partial hydrolysis of the targeted substances with variable bioactivities, and the chemicals used may have an adverse impact on the human body [19]. A low selectivity coefficient restricts the application of activated carbon, and this drawback leads to the loss of valuable products [20]. In contrast, ion exchange technology has the potential to be a more effective method for the decolorization of RD, owing to advantages such as lower loss of yield, higher decolorization efficiency, operational simplicity, and easier regeneration [21].

Currently, RD is mainly decolorized by activated carbon. However, the product recovery rate is relatively low, and the regeneration of activated carbon is difficult. In line with the aforementioned successful application of membrane filtration and ion exchange technology for purification, our objective was to develop a method for removing small molecular impurities (mainly monosaccharides) and pigments from RD using a membrane and resin. The nuclear magnetic resonance spectroscopy (NMR) structure, water solubility, thermal stability, and rheological properties of RD were also characterized to provide a theoretical basis for its application in industrial production.

## 2. Materials and Methods

### 2.1. Materials and Reagents

Yellow dextrin was purchased from Guangrao Lifeng Biological Technology Co., Ltd. (Dongying, China). Three anion exchange resins coded LKA98, D941, and D285 were obtained from Amicogen Biopharm Co., Ltd. (Jining, China). D301R, D301T, and D315 were purchased from the Chemical Plant of Nankai University (Tianjin, China). D280, D380, and D392 were provided by Cangzhou Bonchem Co., Ltd. (Hebei, China). Glucoamylase and α-amylase were obtained from Genencor Bioengineering Co., Ltd. (Wuxi, China). HCl, NaOH, and H_2_SO_4_ were purchased from Sangon Biotech Co., Ltd. (Shanghai, China). The UF membrane (5 kDa pore size) and NF membrane (300–500 Da pore size) were purchased from GE Co., Ltd. (New York, NY, USA). All other reagents were of analytical grade.

### 2.2. Resins and Pretreatment

The physicochemical characteristics of nine resins are listed in Table 1. In accordance with a previous study [18], the pretreatment of resins was carried out as follows: first, to eliminate the adhered impurities, a certain quantity of resin was cleaned with deionized water. Second, in order to remove ethanol-soluble matter, the resins were soaked in 95% ethanol overnight and then washed with distilled water to eliminate traces of ethanol. To further remove the porogenic diluents and monomers that remained inside the pores, the resins were sequentially soaked in 4% HCl and 4% NaOH. Finally, the resins were thoroughly washed with distilled water to neutrality. This pretreatment step was carried out at least thrice. The treated resins were stored in a refrigerator at 4 °C until future use.

### 2.3. Preparation of Crude RD

Crude RD was produced via a reported method with some modifications [1]. Yellow dextrin (50 g) was suspended in 150 mL phosphate buffer (0.05 M, pH 6.0). Then, 0.2% α-amylase (based on the dry weight of yellow dextrin) was added to the solution, which was then reacted at 90 °C for 30 min. Next, the reaction solution was cooled, and the pH was adjusted to 4.5. Based on the dry weight of yellow dextrin, glucoamylase (0.2%) was mixed with the solution, which was then allowed to react at 60 °C for 30 min. Finally, the solution was heated to 90 °C to complete the reaction.

### 2.4. UF and NF of RD

In order to eliminate suspended matter, the solutions, which were adjusted to pH 7.0, were centrifuged at 6000× *g* and 25 °C for 15 min. Then, the supernatant was transferred into membrane devices, comprising a feed tank (minimal operation of 5 L) and a water bath tank for controlling the temperature (Saipu Membrane Technology Development Co., Ltd., Wuxi, China), for further clarification using a UF membrane (5 kDa pore size) and an NF membrane (300–500 Da pore size) in succession. The UF and NF procedures were performed at a temperature of 40 °C, a transmembrane pressure (TMP) of 0.8 MPa, and a velocity of 0.9 m^3^/h. The retentate and permeate were concentrated and stored at 4 °C for subsequent experiments [12].

### 2.5. Static Decolorization Experiments

#### 2.5.1. Effect of Different Resins

To select the most efficient resin, static decolorization tests were performed according to a previously reported study. In a 250 mL Erlenmeyer flask with a stopper, a certain volume of adsorbent, which was equivalent to 5.0 g dry resin, was mixed with 50 mL sample solution (10 mg/mL). The flasks were constantly shaken using a constant temperature oscillator (HZQ-F160, Jingsheng Scientific Instrument Co., Ltd., Shanghai, China) at 170 rpm for 12 h at a constant temperature (30 °C). The resins were then filtered from the solution. The D% and R% values were evaluated to select the optimal resin.

#### 2.5.2. Effects of Time, Temperature, pH, and Initial Sample Concentration on the Decolorization Efficiency

In order to obtain the optimal results, static decolorization tests of different factors were carried out, as summarized below.

Decolorization times of 0, 0.5, 1.0, 2.0, 3.0, 4.0, 5.0, 6.0, 7.0, and 8.0 h were evaluated at a temperature of 30 °C, pH of 7.0, and a solution concentration of 25 mg/mL.

Decolorization temperatures of 20, 25, 30, 35, 40, 45, 50, and 55 °C were assessed with a decolorization time of 4.0 h, pH of 7.0, and a solution concentration of 25 mg/mL.

The effect of pH values of 4.5, 5.0, 5.5, 6.0, 6.5, 7.0, 7.5, 8.0, 8.5, 9.0, and 9.5 was evaluated using a decolorization time of 4.0 h, a solution concentration of 25 mg/mL, and a temperature of 30 °C.

The effect of different solution concentrations of 5, 10, 15, 20, 25, 30, 35, 40, and 45 mg/mL was investigated with a decolorization time of 4.0 h, a pH of 7.0, and a temperature of 30 °C.

### 2.6. Dynamic Decolorization Experiments

Dynamic decolorization tests were carried out using a lab-scale glass column (250 mm × 25 mm) wherein the D285 resin was packed, with a bed volume (BV) of 20 mL. The resin column was thoroughly washed with deionized water at 4.0 BV. For the dynamic decolorization test, an RD solution of 30 mg/mL, adjusted to a pH of 8.0, was loaded onto the resin column at a temperature of 35 °C, and the dynamic leakage curves were evaluated under different flow rates. An auto-partial collector (BSZ-100, Jingke Instrument Co., Ltd., Shanghai, China) was used to collect the effluent of RD. The D% and R% were used to evaluate the optimal feeding volume and flow rate.

### 2.7. Analytical Measurements

#### 2.7.1. Measurement of Turbidity

A ratio turbidimeter (Hach, Loveland, CO, USA) was used for the measurement of the turbidities of the retentate and permeate after appropriate dilution whenever necessary [22].

#### 2.7.2. Measurement of Decolorization Ratio

In accordance with a previously reported method, the data for the D% were collected as follows [23]: samples were diluted to a proper concentration with deionized water before the absorbance (Abs) of each solution was measured at 420 nm using UV–Vis spectroscopy (UV-1800, Aoyi Instruments Shanghai Co., Ltd., Shanghai, China). The D% of RD was calculated as follows:

(1) $$D\%=\frac{A_{D0}-A_{D1}}{A_{D0}}\times 100\%$$

where *A_D_*_0_ and *A_D_*_1_ are the absorbances at 420 nm of the solutions of RD before and after treatment, respectively. *D*% is the decolorization ratio of RD.

#### 2.7.3. Measurement of Recovery Ratio

The RD content was evaluated by HPLC. Before the concentration of RD was investigated, the samples were pretreated with a membrane filter (0.22 μm). HPLC experiments were carried out with an Agilent 1260 chromatography (Beijing, China), which was equipped with a G7162A refractive index detector and a SugarPak 1 column (300 mm × 6.5 mm). The mobile phase was ultrapure water with a flow rate of 0.4 mL/min and a column temperature of 85 °C. The *R*% of RD was calculated as follows:

(2) $$R\%=\frac{M_{1}}{M_{0}}\times 100\%$$

where *M*_0_ and *M*_1_ are the concentrations of RD before and after treatment, respectively. *R*% is the recovery ratio of RD.

#### 2.7.4. Measurement of Competitive Coefficient

*A_D_*_0_, *A_D_*_1_, *M*_0_, and *M_1_* were determined as in Section 2.7.2 Measurement of Decolorization Ratio, Section 2.7.3 Measurement of Recovery Ratio. Distribution coefficient (*K_d_*) and competitive coefficient (*K_c_*) were calculated by the following equations [24]:

(3) $$K_{d1}=\frac{V(A_{D0}-A_{D1})}{W\times A_{D1}}$$

(4) $$K_{d2}=\frac{V(M_{0}-M_{1})}{W\times M_{1}}$$

(5) $$K_{c}=\frac{K_{d1}}{K_{d2}}$$

where *W* is the weight of the resins (g), *V* is the volume of solution (L), Q is the amount (mg/g), *K_d1_* and *K_d2_* are the distribution coefficient of pigment and RD on resins, respectively, and *K_c_* is the competitive coefficient of RD.

### 2.8. Comparison of RD before and after Decolorization by D285 Resin

The UV–Vis full-wave spectrometer and Fourier transform infrared spectroscopy (FT-IR) (Nicolet iS10, Precision Instrument Co., Ltd., Shanghai, China) were used to characterize RD before and after treatment according to previously reported methods [18]. High-performance gel permeation chromatography (HPGPC) was used to assess the molecular weight distribution of RD. The HPLC system (Waters 1525, Milford, MA, USA) was equipped with an Ultrahydrogel TM Linear column (300 mm × 7.8 mm, Waters, Milford, MA, USA) and a refractive index detector (RID). The mobile phase was a sodium nitrite solution (0.1 M) with a flow rate of 0.8 mL/min at 35 °C. Calibration was performed using standard dextrans with different molecular weights. In addition, a digital camera was used to photograph the RD solutions before and after decolorization.

### 2.9. Comparison of Different Decolorization Approaches

The decolorization efficiency of the resin was further compared with that of traditional methods, and treatments of RD using activated carbon and H_2_O_2_ were carried out as follows [20]. The RD solution used in dynamic decolorization was used in subsequent tests. In order to evaluate the decolorization efficiency of H_2_O_2_, 30% H_2_O_2_ (50 mL) and the RD solution (50 mL) were mixed and continually shaken at 35 °C for 6.0 h. Decolorization experiments of activated carbon were carried out based on a previously reported method: the RD solution was loaded onto an activated carbon column (equal to 20 g dried matter) three times. The molecular weight, D%, and R% of RD were assessed.

### 2.10. NMR Spectroscopy

To reduce the effect of water, the RD sample (25.0 mg) was exchanged twice with D_2_O (500 μL). The pretreated RD was dissolved in D_2_O at a concentration of 50 mg/mL and tested according to a method used in a previous study [2]. The ^1^H NMR spectra, recorded on a 400 M AVANCE III NMR spectrometer (Bruker, Switzerland) at 60 °C, were collected in 64 individual scans with a delay of 1 s. Tetramethylsilane was used as an internal reference at 0 ppm.

### 2.11. Characterization of RD

RD was characterized by comparison with commercially available corn starch (Tiancheng Corn Development Co., Ltd., Siping, China) and RDs (RD-1 and RD-2, obtained from Shandong Bailong Chuangyuan Bio-Tech Co., Ltd., Dezhou, China and Shandong Longlive Bio-Tech Co., Ltd., Dezhou, China, respectively). The preparation method of crude RD-1 and RD-2 is similar to that of RD, but the method of decolorization and separation is different. RD-1 and RD-2 were decolorized with activated carbon and separated by chromatographic separation to remove monosaccharides.

#### 2.11.1. Scanning Electron Microscopy (SEM)

Micrographs were captured using an SU8100 scanning electron microscope (Hitachi, Tokyo, Japan). The freeze-dried samples were evenly distributed on the sample stage with a double adhesive tape and plated with a conductive gold layer (approximately 10 nm thick). The micrographs were obtained under low vacuum at an accelerating potential of 15 kV and were used to observe any damage to the sample surface.

#### 2.11.2. Water Solubility

The water solubility of the dextrins was calculated using a previously reported protocol [25]. The freeze-dried samples (100.0 mg) were mixed well with water (900 μL). The solution was centrifuged at 12,000× *g* for 3 min. The supernatant was collected and dried at 40 °C for 6.0 h, followed by drying at 110 °C in an oven for 3.0 h. The percentage of dissolved substance with respect to the total weight was used to evaluate water solubility.

#### 2.11.3. Thermal Properties

Thermogravimetric analysis (TGA) of dextrin samples was performed using a TG analyzer (TGA2, METTLER TOLEDO, Columbus, OH, USA) to determine the thermal stability. Dextrin samples (3–5 mg) were placed in the weighed aluminum sample cell. The samples were heated from 25 to 400 °C at 10 °C/min, with a nitrogen flow rate of 50 mL/min, and the mass loss was recorded.

#### 2.11.4. Rheological Properties

The rheological properties of the dextrins were determined using DHR-3 (TA Instruments, New Castle, DE, USA). A parallel plate (40 mm diameter, 1 mm gap) with a controlled temperature (25 °C) was used. The shear rate was varied from 0.1 to 100 s^−1^.

### 2.12. Statistical Analysis

SPSS for Windows, Version 16.0 (SPSS, Chicago, IL, USA) was used to carry out ANOVA followed by Duncan’s multiple-range tests. The data are presented as the mean ± SD, and *p* < 0.05 was regarded as statistically significant. Each experiment was performed thrice.

## 3. Results and Discussion

### 3.1. UF and NF

**Figure 1:**
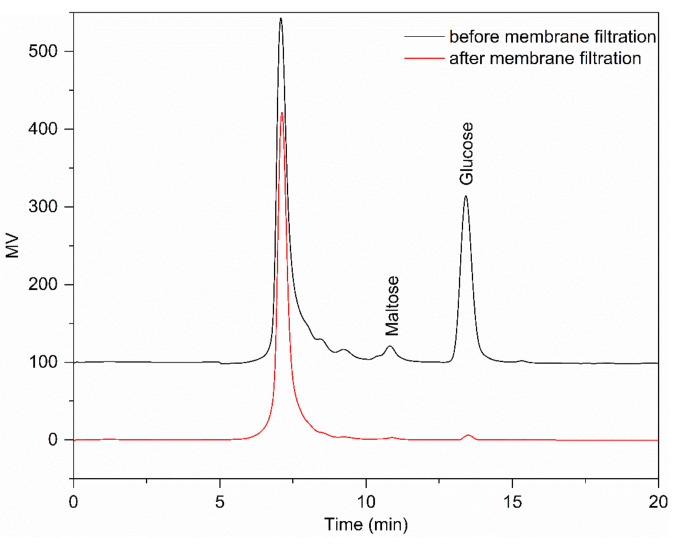
The HPLC chromatograms of resistant dextrin (RD) before and after filtration using an ultrafiltration (UF) membrane (5 kDa pore size) and a nanofiltration (NF) membrane (300–500 Da pore size) in succession.

Figure 1 shows that the monosaccharides and disaccharides were almost completely removed by NF. It can be seen from Table 2 that after UF and NF, RD had a small proportion of monosaccharides and disaccharides compared with RD-1 and RD-2. Therefore, membrane filtration is an effective way to achieve separation and purification of RD. Interestingly, the monosaccharides presented higher levels than saccharides. The reason could be that the content of monosaccharides in crude RD was far more than disaccharides; increasing the frequency of NF may reduce the content of monosaccharide, but it would cause the loss of RD. It is evident from Table 3 that suspended solids in crude RD were completely removed by UF, and the resulting product had negligible turbidity. The remarkable decrease in turbidity, as high as 98.06%, was due to the removal of high-molecular-weight impurities and fine particulates. Permeate turbidity shows that effective clarification can be achieved by UF, as the process obtained a clear permeate and reduced the fouling for the following step. Susanto et al. [13] found that ultrafiltration (UF) followed by ion exchange (IE) could produce colorless liquid sugar, and this UF–IE configuration showed a better performance than both the IE-UF and UF/IE alone because UF eased the burden of IE resins. Meanwhile, half of the pigments were removed through membrane filtration. However, many pigments were retained, and further processing with an anion exchange resin was required to obtain high-quality RD. Similar results were obtained in other studies [14]. It was found that the maximum D% was not significantly different when the pore size of the membrane was lower than a certain value.

### 3.2. Static Decolorization Test of Ten Resins

**Figure 2:**
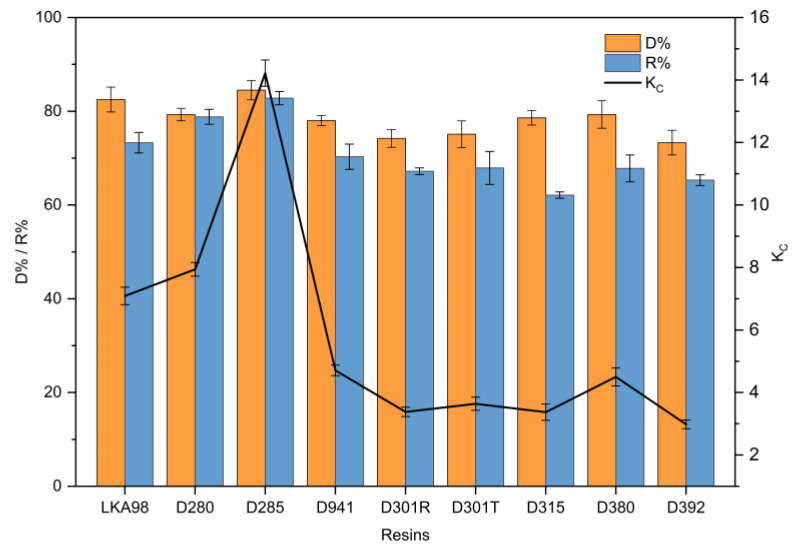
Comparison of resistant dextrin (RD) recovery ratio (R%) and decolorization ratio (D%) of nine different anion exchange resins in static decolorization experiments.

Many factors, such as pore diameter, surface area, hydrogen bonding interactions, surface electrical properties, and functional groups, affect the decolorization efficiency. For a higher decolorization efficiency, the optimal resin should have a strong adsorption capacity for pigments and a weak affinity for RD. It can be seen from Figure 2 that the D% was over 70% among all the anion exchange resins. Notably, strongly basic anion exchange resins exhibited superior decolorization efficiency among all the resins. This was probably because the pH did not have a significant influence on the number of positively charged functional groups of the strongly basic anion exchange resins. However, the pH determines the number of positive charges of the tertiary amines possessed by the weakly basic exchangers [26]. In addition, the negative charges of Cl^−^ of the counter ion were entirely dissociated from the functional groups of the strongly basic resins [27]. Interestingly, the strongly basic anion exchange resins exhibited a higher R% than the weakly basic anion exchange resins. The reason for this is that pigments, which occupy more active sites of resins, are mostly aromatic compounds with relatively strong negative charges. The D% of LKA98 and D285 resins was significantly higher than that of other resins (*p* < 0.05), and the R% of D285 resin was the highest among all resins (*p* < 0.05). Compared with all other tested resins, the D285 resin exhibited a superior D% and R%, owing to its higher total exchange capacity and stronger affinity towards pigments. Meanwhile, the highest K_c_ also indicated that D285 resin had better selectivity and could better adsorb pigments. Thus, the D285 resin was selected for subsequent experiments.

### 3.3. Effect of Different Factors

#### 3.3.1. Effect of Time

**Figure 3:**
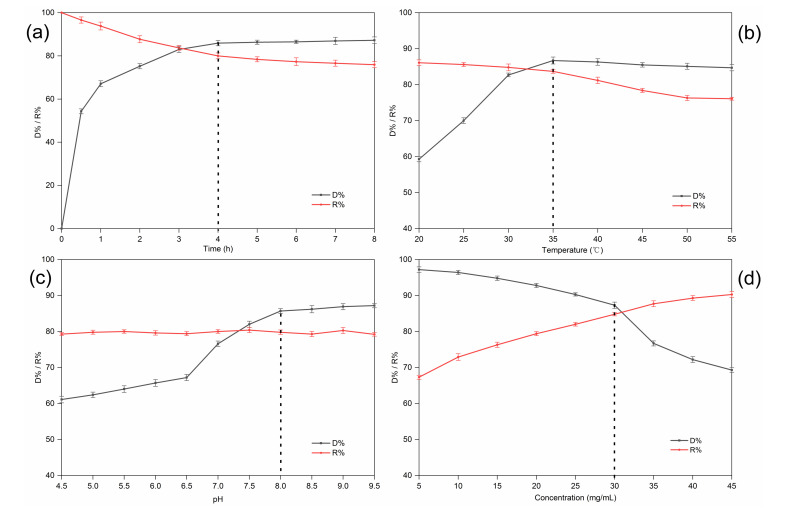
Effect of time (**a**), temperature (**b**), pH (**c**), and sample concentration (**d**) on the decolorization ratio (D%) and recovery ratio (R%) of resistant dextrin (RD) on D285 resin in static decolorization experiments.

The decolorization time had a significant impact on the D% and R% of RD. Extending the contact time could promote the adsorption of pigments. However, a higher amount of RD was lost because of the longer decolorization time. The results are shown in Figure 3a. When the decolorization time was extended, the D% of the D285 resin increased, but there was no clear distinction among samples treated from 4.0 to 8.0 h (*p* > 0.05). This is because the D285 resin was saturated after approximately 4.0 h. Moreover, the RD R% initially declined dramatically with an increase in the decolorization time (*p* < 0.05). However, the decrease became insignificant (*p* > 0.05) and less dramatic after 4.0 h. It has been proposed that the initial rapid adsorption occurred because of the high diffusion of solute molecules into the pores of the resin, while the following slow adsorption process was due to the high intraparticle mass transfer resistance inside the resin [28]. Thus, a decolorization time of 4.0 h was chosen for the subsequent experiments. Studies on the adsorption kinetics of many substances have confirmed this result [20,29].

#### 3.3.2. Effect of Temperature

Figure 3b indicates that the D% dramatically increased from 20 to 35 °C (*p* < 0.05). However, there was no clear change in the D% when the temperature exceeded 35 °C. Moreover, the R% of RD significantly decreased with increasing temperature between 35 and 55 °C (*p* < 0.05). On one hand, the mobility of the molecules increased due to the increase in kinetic energy, and a higher temperature promoted intraparticle diffusion of the adsorbate [30]. On the other hand, the increase in temperature made the boundary layer around the resin beads thinner; hence, the mass transfer resistance through the boundary layer decreased [31]. The increase in the thermal movement of the solute could also lead to the desorption of the adsorbed substances from the resin, causing a decrease in the resin adsorption capacity. Although our results suggest that decolorization is favored at higher temperatures, other studies have suggested that adsorption is an exothermic process and, hence, low temperatures are beneficial for decolorization [32]. In our study, an operating temperature of 35 °C was selected for subsequent experiments.

#### 3.3.3. Effect of pH

Figure 3c shows that the D% went up with increasing pH in the beginning (*p* < 0.05). and finally reached an equilibrium at approximately pH 8.0. Generally speaking, the decolorization efficiency in an alkaline solution was higher than that in neutral or acidic solutions. There are two assumed mechanisms (ion exchange and adsorption) for the uptake of pigments into the resin beads. On the one hand, the interactions of hydrogen bonds would be reduced due to fewer pigments and more protonation in the alkaline solution. On the other hand, the polyphenol hydroxyl groups of the pigments would dissociate more easily into negative ions in solutions of high pH and, thus, be adsorbed by the resin [33,34]. However, the R% of RD did not change significantly with increasing pH (*p* > 0.05). Interestingly, Liu et al. found that pigments were adsorbed more easily by macroporous resins than by anion exchange resins at a relatively low pH. This is because hydrogen bonding has a significant influence on the adsorption of macroporous resins. Hydrogen bonding interactions decrease at higher pH values, resulting in a decreased adsorption capacity of such resins [28]. Taking both cases into consideration, we selected pH 8.0 for the subsequent experiments.

#### 3.3.4. Effect of Solution Concentration

The number of functional groups determines the resin capacity. Generally, for a fixed amount of selected resin, the active sites available at high solution concentrations are less than those at low initial concentrations [35]. However, ratios higher than a certain value did not improve the decolorization efficiency linearly, indicating that the availability of the adsorption surface was no longer the limiting factor in the process. Moreover, a higher amount of RD was adsorbed and lost when the sample concentration was low. Figure 3d shows that the D% increased (*p* < 0.05) and the R% decreased (*p* < 0.05) simultaneously with increasing sample concentration. However, the D% dropped more significantly after the sample concentration reached 30 mg/mL. Therefore, a concentration of 30 mg/mL was selected for subsequent experiments. Similar results were reported for the adsorption of colored impurities in soybean oligosaccharides extracted from sweet slurry using adsorption resins [32].

### 3.4. Dynamic Decolorization Experiments on the D285 Resin

In the dynamic adsorption experiment, the resin was loaded until the outlet solute concentration exceeded a certain threshold, and this was defined as a breakthrough point [20]. The breakthrough point indicates a decline in the number of active sites and leakage of the targeted substance from the bed. Therefore, to select the appropriate parameters (loading volume and flow rate), it is vital to establish a dynamic leakage curve.

**Figure 4:**
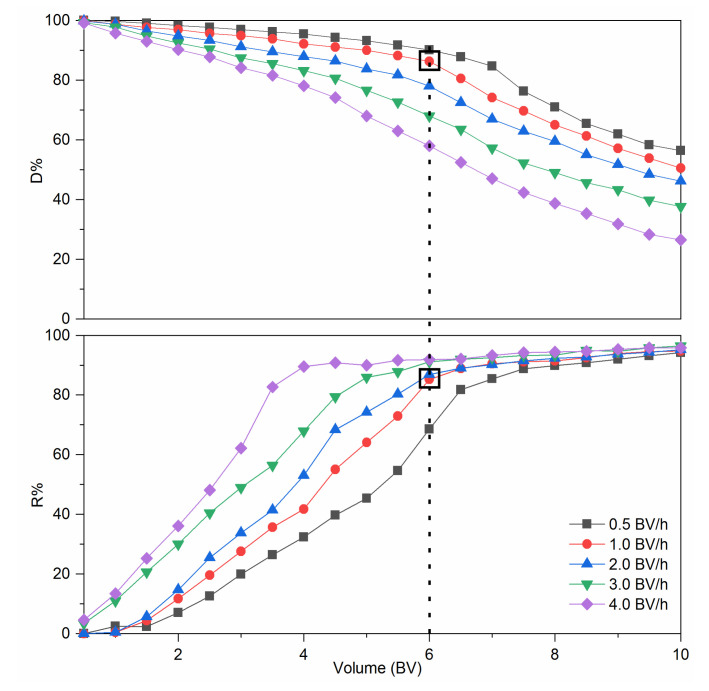
Dynamic leakage curves of colored impurities and resistant dextrin (RD) on D285 resin.

Figure 4 shows the dynamic leakage curves. At a lower flow rate, the resin bed exhibited better decolorization performance. This result could be explained by the slower flow rate which allowed the pigments and RD to have more time to interact with the functional groups of the D285 resin [36]. As the flow rate increased, a higher R% and lower D% of RD were simultaneously observed. However, a lower flow rate is disadvantageous in industrial production because of the extension of the working time. In order to better show the difference in the decolorization effect under different dynamic decolorization conditions, we introduced a new parameter, namely, the separation coefficient *K_c_*. It can be clearly seen from Table 4 that when the flow rate was 1.0 BV/h and the outflow volume was 6.0 BV, *K_c_* is the largest. Figure 4 shows that when the flow rate was 1.0 BV/h, the D% decreased indistinctively (*p* > 0.05) from 0.5 to 6.0 BV. Meanwhile, R% increased insignificantly (*p* > 0.05) when outflow volume exceeded 6.0 BV. In addition, when the outflow volume was 6.0 BV/h, the D% under 1.0 BV/h was evidently higher than that under 2.0, 3.0, and 4.0 BV/h (*p* < 0.05) and the R% under 1.0 BV/h was markedly higher that under 0.5 BV/h (*p* < 0.05). Therefore, the optimal processing volume and flow rate were determined to be 6.0 BV and 1.0 BV/h, respectively.

### 3.5. Characterization of RD Treated by the D285 Resin

**Figure 5:**
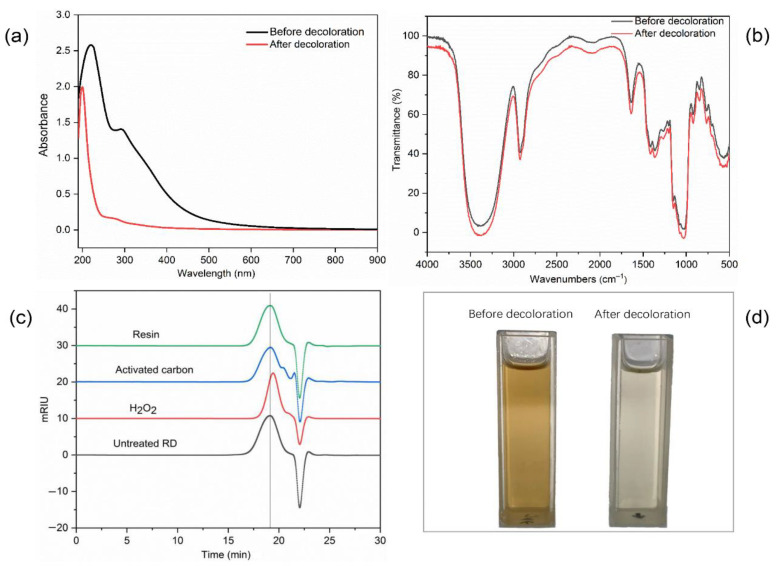
The UV–Vis spectra (**a**), Fourier transform infrared spectroscopy spectra (**b**), molecular weights (**c**), and photographs (**d**) of resistant dextrin (RD) before and after decolorization using D285 resin.

To assess the decolorization efficiency of the D285 resin, RD was characterized using digital photography, FT-IR spectroscopy, and UV–Vis spectroscopy before and after treatment. Figure 5a shows that the absorbance at 300–600 nm significantly decreased after decolorization, indicating that most of the colored impurities in RD were removed. Figure 5b shows that there was no clear change after treatment with the D285 resin, indicating that the functional groups and chemical structure of RD were adequately retained during the purification process. As shown in Figure 5c, there was no dramatic difference in the molecular weight after decolorization by the D285 resin. As shown in Figure 5d, the comparison of photography demonstrated the excellent efficiency of the D285 resin. Compared with the dark brown solution before treatment, the solution was nearly colorless after the decolorization process with the resin. Therefore, D285 resin could be a preferred material for the purification of RD.

### 3.6. Comparison of Different Decolorization Methods

As summarized in Table 5, the R%, D%, and molecular weight of RD after decolorization using the anion exchange resin were 86.26 ± 0.63%, 85.23 ± 0.42%, and 2.71 ± 0.09 kDa, respectively. The decolorization efficiency of the D285 resin was higher than those of activated carbon and H_2_O_2_. Furthermore, the molecular weights of RD after decolorization with activated carbon or using the present method were not significantly different from those of the untreated sample. The results indicated that anion exchange technology could be a potential procedure for the decolorization of RD in industry.

### 3.7. NMR Spectroscopy

**Figure 6:**
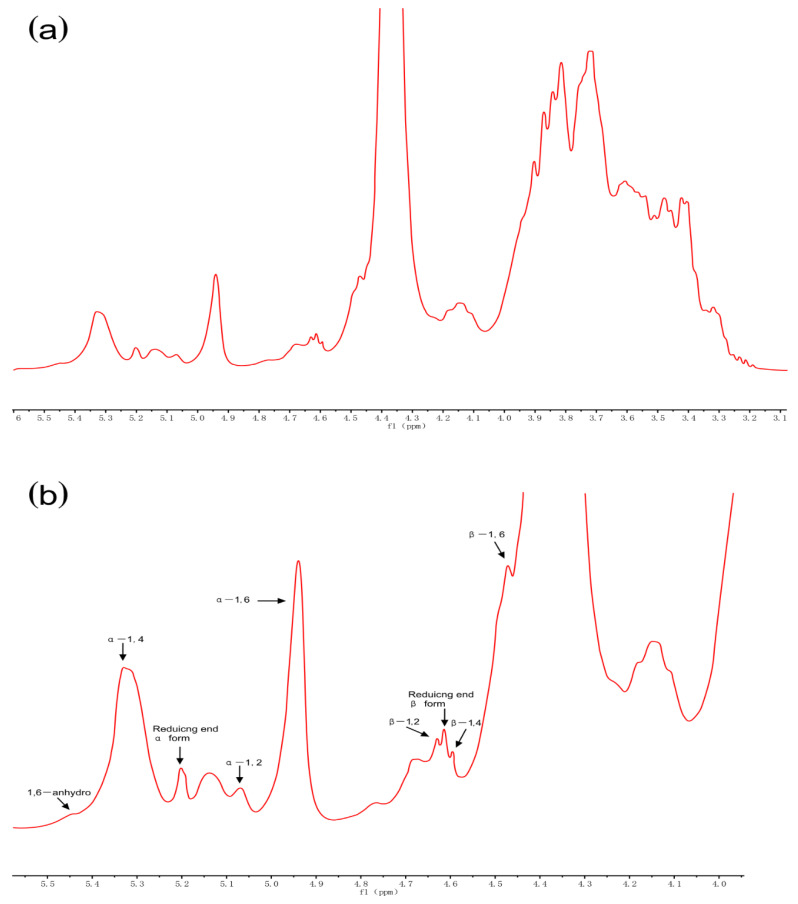
^1^H nuclear magnetic resonance spectra (recorded at 60 °C) of resistant dextrin (RD) (**a**) and its expanded region (**b**) purified using membrane filtration and anion exchange resin decolorization.

NMR spectroscopy was used to analyze the glycosidic linkages of RD. Anomeric protons in areas ranging from 4.4 to 5.5 ppm were all separated in the ^1^H NMR spectrum (Figure 6) and new peaks (5.45, 5.07, 4.55–4.65, and 4.47 ppm) were observed with respect to those of maize starch and maltodextrin [6]. These new linkages (1,6-anhydro, α-1,2, β-1,2, β-1,4, and β-1,6 bonds) were formed during dextrinization, and the branched structure of RD is considered responsible for its increased resistance to digestion. The reason for the formation of these new bonds is that 1,6 anhydro-β-D-glucopyranose formed in the intramolecular and oxocarbonium ions or the free radicals attacked the adjacent hydroxyl groups. Chen et al. found that the reaction conditions significantly affected the glycosidic linkages structure of pyrodextrin and these newly formed branched bonds could not be dissociated by digestive enzymes owing to steric hindrance [6].

### 3.8. Characterization of RD

#### 3.8.1. Scanning Electron Micrographs

**Figure 7:**
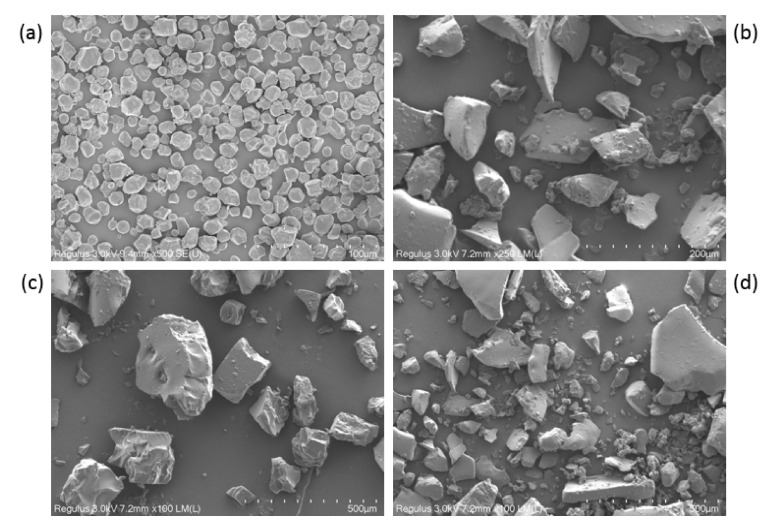
Scanning electron images of starch and the dextrins: (**a**) native starch, (**b**) resistant dextrin (RD), (**c**) RD-1, and (**d**) RD-2.

The size and surface morphology of the native starch, RD, RD-1, and RD-2 granules were compared using an SEM instrument. The SEM images of all the samples are displayed in Figure 7. The size and shape of the dextrin granules were obviously different from those of native starch. The shape of the native starch granules was oval or spherical (Figure 7a), and no cracks were observed on the surface. However, no intact starch granules were observed in the dextrin samples, and the starch granules became fragments due to severe acid–thermal hydrolysis. This indicates that most of the starch granules were damaged during dextrinization (Figure 7b–d). Bai et al. studied the structural changes upon thermal treatment during the conversion of native starch to pyrodextrin [37]. It was found that the intensity of the diffraction peak decreased with increasing heating time. This could be explained by the hydrolysis of the amorphous regions and partial destruction of the crystalline flakes, which resulted in the loss of the periodic lamellar structure. The acid mainly hydrolyzes the amorphous regions of the starch granules. It is likely that the periodic lamellar structure will no longer be formed after a large number of glycosidic bonds are dissociated and the crystalline lamellae are destroyed.

#### 3.8.2. Water Solubility

Heating time, pH, and temperature have a significant influence on the solubility of dextrins. In our study, RD had comparable solubility (99.14%) compared to that of RD-1 and RD-2 (98.68% and 98.85%, respectively). During dextrinization, the starch molecules are hydrolyzed under acidic conditions and heat, resulting in a decreased molecular size. Han et al. [25] demonstrated that the product of starch treated by a similar process had a fairly high solubility. The good solubility of RD is the prerequisite for its application in food, medicine, and other fields.

#### 3.8.3. Thermal Properties

**Figure 8:**
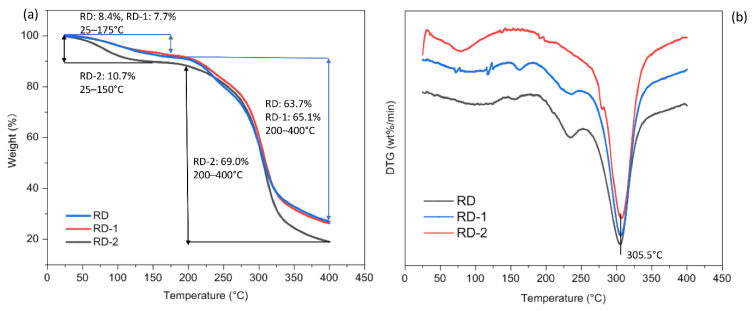
The thermogravimetric analysis (TGA) (**a**) and derivative thermogravimetric (DTG) (**b**) curves of resistant dextrin (RD), RD-1, and RD-2 that were heated from 25 to 400 °C at 10 °C/min.

TGA measures the change in sample mass with respect to temperature and can be used to determine the thermal stability of a sample. The derivative thermogravimetric (DTG) curves, used to evaluate the rate of mass loss, are superior for estimating the thermal transition process. Figure 8a suggests that the initial mass loss of RD (8.4%) and RD-1 (7.7%) between 25 and 175 °C, and RD-2 (10.7%) between 25 and 150 °C might be due to moisture loss, suggesting that the dextrins were not really anhydrous. RD-2 lost more mass during this phase compared to those of RD and RD-1. This may be due to the relatively low temperature during the preparation of RD-2, which caused this significant weight loss. In addition, it can be seen from the TGA curve that all samples were relatively stable below 200 °C. During the degradation phase, ranging from 200 to 400 °C, the mass of all samples was greatly reduced (RD: 63.7%, RD-1: 65.1%, RD-2: 69.0%). A sharp peak was observed on the DTG curve (Figure 8b) at 305.5 °C, marking the degradation temperature (Td). Thus, to avoid damaging the structural integrity, all samples should not be subjected to temperatures above 200 °C. The potential industrial application of dextrins is closely related to its thermal behavior, and a high Td would mean that dextrins can be used in food processing systems involving temperatures below 200 °C.

#### 3.8.4. Rheological Properties

**Figure 9:**
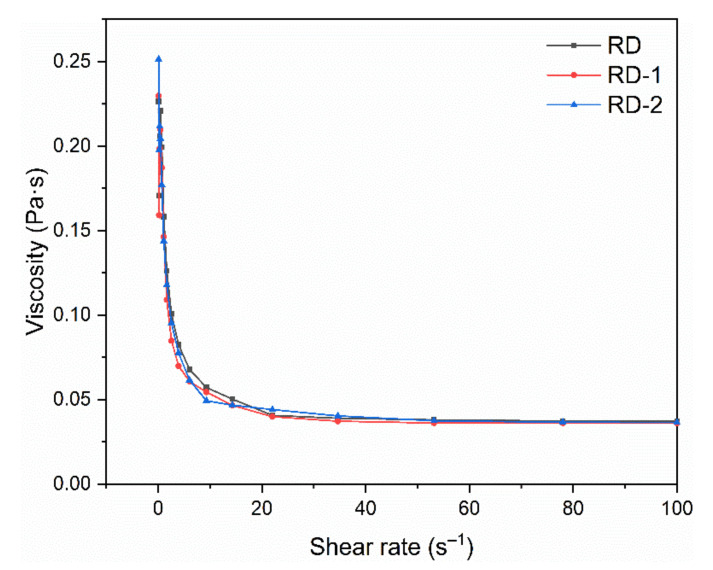
Rheological behavior of resistant dextrin (RD), RD-1, and RD-2 against shear rates ranging from 0.1 to 100 s^−1^ using a parallel plate (40 mm diameter, 1 mm gap) with controlled temperature (25 °C).

Figure 9 shows the apparent viscosity of dextrins at shear rates ranging from 0.1 to 100 s^−1^. All the samples showed good pseudoplastic rheological properties; the apparent viscosity decreased with an increase in the shear rate. The overall viscosity of dextrins was relatively low, and this property is particularly important for their application in the beverage industry. Fibersol-2 (Matsutani Chemical Industry Co., Ltd., Hyogo, Japan) is a commercially available RD that has been used in a variety of beverage products. Zhang et al. added RD to red and rice wine, and found that the organoleptic quality of wines improved due to the combination of RD with polyphenols [38].

## 4. Conclusions

The purification of RD was successfully achieved in this study. After centrifugation and microfiltration, the pretreated RD was clarified using a 5 kDa pore size UF membrane. This resulted in the complete removal of turbidity, owing to the suitable pore size. In addition, the monosaccharides and disaccharides were almost completely removed by NF. Among the various anion exchange resins tested, the D285 resin exhibited the best D% and R% of RD. Static decolorization experiments under varying conditions were used to determine the optimal decolorization parameters, and a dynamic decolorization experiment was used to determine the proper flow rate and volume of solution. The present method for the purification of RD is superior to the conventional approaches owing to its high efficiency. Thus, this method is a promising approach for the mass production of RD. The water solubility, thermal stability, and rheological properties of RD provide a theoretical basis for industrial applications.

## Figures and Tables

**Table 1:** Physicochemical characteristics of the tested resins.

Resin	Appearance	Skeleton Material	Functional Group	Wet True Density (g/mL)	Total Exchange Capacity (dry, mmol/g)	Sized Bead Content after Grinding (%)
LKA98	Canary yellow	Acrylic acid	-N(CH_3_)_3_	1.03–1.10	≥4.6	≥95
D280	Light yellow	Styrene	-N(CH_3_)_3_	1.06–1.11	≥3.0	≥90
D285	Canary yellow	Styrene	-N(CH_3_)_3_	1.03–1.10	≥6.4	≥95
D941	Milky white	Acrylic acid	-N(CH_3_)_2_	1.07–1.12	≥5.0	≥95
D301R	Slight yellow	Styrene	-N(CH_3_)_2_	1.03–1.07	≥4.6	≥95
D301T	Slight yellow	Styrene	-N(CH_3_)_2_	1.03–1.07	≥4.6	≥95
D380	Slight yellow	Acrylic acid	-NH_2_	1.03–1.10	≥6.4	≥90
D392	Yellow	Styrene	-NH_2_	1.05–1.10	≥4.6	≥95
D315	Slight yellow	Acrylic acid	-NH_2_	1.06–1.12	≥6.4	≥95

**Table 2:** The proportion of degree of polymerization (DP) = 1 (namely, glucose), DP = 2 (namely, maltose), and DP ≥ 3 (namely, oligosaccharides with a DP greater than three) of RD, RD-1, and RD-2.

Samples	DP = 1	DP = 2	DP ≥ 3
RD	4.34%	1.24%	94.42%
RD-1	2.45%	5.53%	92.02%
RD-2	3.01%	6.05%	90.94%

**Table 3:** Absorbance and turbidity of the resistant dextrin (RD) solution before and after filtration using a 5 kDa pore size ultrafiltration (UF) membrane and 300–500 Da pore size nanofiltration (NF) membrane.

Parameter	Untreated Sample	Treated Sample
Color (A_420_)	0.821 ± 0.029	0.433 ± 0.014
Turbidity (NTU)	139.20 ± 3.01	2.70 ± 0.18

**Table 4:** The competitive coefficient (K_c_) of RD under different flow rates and volumes in dynamic decolorization experiments.

Volume (BV)	Flow Rate (BV/h)				
	0.5	1.0	2.0	3.0	4.0
0.5	0.00	0.00	0.00	2.41	2.67
1.0	3.34	0.15	0.19	2.74	1.78
1.5	1.29	0.95	0.85	2.44	2.32
2.0	2.27	2.10	1.63	2.73	2.75
2.5	3.04	2.70	2.44	3.35	3.52
3.0	3.98	3.61	2.75	3.56	4.73
3.5	4.56	4.37	3.18	4.11	11.63
4.0	5.07	4.39	4.37	5.72	17.08
4.5	5.56	6.50	7.33	8.89	16.25
5.0	5.89	8.49	8.03	11.37	11.44
5.5	6.88	10.68	9.98	11.12	11.49
6.0	10.45	19.44	13.15	13.08	9.91
6.5	16.99	18.35	12.37	12.30	8.44
7.0	17.44	15.68	11.12	10.52	8.41
7.5	14.40	14.07	11.24	10.04	8.41
8.0	12.68	12.13	11.12	9.20	7.83
8.5	11.35	12.22	10.03	10.90	7.24
9.0	11.54	13.22	10.49	9.49	7.14
9.5	12.02	13.12	10.47	10.81	7.08
10.0	13.69	12.23	11.71	11.88	6.57

**Table 5:** Comparison of decolorization effect of H_2_O_2_, activated carbon, and D285 resin values, with different letters in the same column indicating significant differences (*p* < 0.05).

Methods	R%	D%	Molecular Weight (kDa)	
			Before Decolorization	After Decolorization
H_2_O_2_	36.70 ± 1.27 ^C^	72.65 ± 1.17 ^B^	2.69 ± 0.13	1.92 ± 0.15 ^B^
Activated carbon	54.10 ± 1.04 ^B^	85.50 ± 1.90 ^A^		2.66 ± 0.17 ^A^
Present method	85.23 ± 0.42 ^A^	86.26 ± 0.63 ^A^		2.71 ± 0.09 ^A^
